# Supplementary material for: Dynamic transcriptomic profiles of zebrafish gills in response to zinc supplementation
Source: BMC Genomics. 2010 Oct 11;11:553. doi: 10.1186/1471-2164-11-553 (PMC3091702; doi:10.1186/1471-2164-11-553)
Supplement: Additional file 2 — Interactive Direct Interaction Network representing the molecular interactions between zinc, copper, iron, calcium and proteins encoded by transcripts changed by zinc supplementation. Mini web-site containing index.html and hyperlinked pages in subdirectory describing a Direct Interaction Network automatically generated based on curated interactions contained within the proprietary PathwayArchitect database. Ovals represent proteins and the circles symbolize metal ions. Objects are coloured by their abundance in zebrafish at the time-point they were significantly different from the control is a scale from -4 fold (dark green) to +4 fold (dark red). Where significant differences were found at more than one time-point, the colour overlay shows expression at the first instance. Dark blue squares denote 'binding', and light blue squares 'expression'; green squares stand for 'regulation', green diamonds for 'metabolism', and green circles for 'promoter binding'. Arrow heads indicate directionality of the interaction where annotated. All nodes and edges can be further interrogated by selecting the relative area of the image. [file 1471-2164-11-553-S2.zip › PathwayArchitect Zn xs DIN/85689.html]

# PROTEIN: GATA2

|  |  |
| --- | --- |
| Name | GATA2 |
| Type | PROTEIN |
| Description | GATA binding protein 2 |
| Note | The GATA family of transcription factors, which contain zinc fingers in their DNA binding domain, have emerged as candidate regulators of gene expression in hematopoietic cells (Tsai et al., 1994). GATA1 (MIM 305371) is essential for normal primitive and definitive erythropoiesis and is expressed at high levels in erythroid cells, mast cells, and megakaryocytes. GATA2 is expressed in hematopoietic progenitors, including early erythroid cells, mast cells, and megakaryocytes, and also in nonhematopoietic embryonic stem cells. In chicken erythroid progenitors, forced expression of GATA2 promotes proliferation at the expense of differentiation (Briegel et al., 1993). GATA3 (MIM 131320) expression is restricted to T-lymphoid cells and some nonhematopoietic cell types, including embryonic stem cells.[supplied by OMIM] |
| Alias | MGC129339 |
|  | NFE1B |
|  | Gata-2 |
|  | GATA2 |
|  | GATA-bindning protein 2 |
|  | Gata2 |
|  | GATA-binding protein 2 |
|  | MGC2306 |


---

|  |  |
| --- | --- |
| GO Component | nucleus |


---

|  |  |
| --- | --- |
| GO ID | GO:0030182 |
|  | GO:0005634 |
|  | GO:0003677 |
|  | GO:0006355 |
|  | GO:0006366 |
|  | GO:0050766 |
|  | GO:0046872 |
|  | GO:0006350 |
|  | GO:0003700 |
|  | GO:0006909 |


---

|  |  |
| --- | --- |
| MIM | MIM:137295 |


---

|  |  |
| --- | --- |
| Connectivity | 200 |


---

|  |  |
| --- | --- |
| Entrez ID | 14461 |
|  | 2624 |
|  | 25159 |


---

|  |  |
| --- | --- |
| Agilent ID | A\_44\_P403866 |
|  | A\_14\_P122980 |
|  | A\_14\_P110929 |
|  | A\_53\_P137369 |
|  | A\_53\_P160442 |
|  | A\_52\_P233441 |
|  | A\_53\_P150573 |
|  | A\_51\_P516625 |
|  | A\_51\_P516626 |
|  | A\_24\_P165998 |
|  | A\_43\_P14273 |
|  | A\_23\_P110022 |


---

|  |  |
| --- | --- |
| Cellular Localization | Nucleus |
|  | Organelle |
|  | Cell |


---

|  |  |
| --- | --- |
| Pathway | Zn xs inventory |
|  | Zn xs DIN |


---

|  |  |
| --- | --- |
| GO Process | neuron differentiation |
|  | phagocytosis |
|  | regulation of transcription, DNA-dependent |
|  | positive regulation of phagocytosis |
|  | transcription |
|  | transcription from RNA polymerase II promoter |


---

|  |  |
| --- | --- |
| UniGene | Rn.34322 |
|  | Hs.367725 |
|  | Mm.272747 |


---

|  |  |
| --- | --- |
| Affymetrix Probeset ID | 102789\_at |
|  | 1071\_at |
|  | 1072\_g\_at |
|  | 1368297\_at |
|  | 1428816\_a\_at |
|  | 1450333\_a\_at |
|  | 161820\_f\_at |
|  | 203\_at |
|  | 207954\_at |
|  | 209710\_at |
|  | 210358\_3p\_x\_at |
|  | 210358\_x\_at |
|  | 37194\_at |
|  | AB000096\_at |
|  | AB000096\_g\_at |
|  | g12803464\_3p\_a\_at |
|  | g12803464\_3p\_x\_at |
|  | g4503926\_3p\_a\_at |
|  | g4503926\_3p\_at |
|  | Hs.760.1.S2\_3p\_at |
|  | M68891\_at |
|  | M77810\_at |
|  | rc\_AA899775\_at |
|  | RC\_H01824\_f\_at |
|  | RC\_R78436\_f\_at |
|  | RC\_R78436\_i\_at |
|  | TC32040\_s\_at |


---

|  |  |
| --- | --- |
| GO Function | transcription factor activity |
|  | DNA binding |
|  | metal ion binding |


---

|  |  |
| --- | --- |
| Nucleotide | BC002557 |
|  | NM\_033442 |
|  | BX647499 |
|  | AF345897 |
|  | BT006671 |
|  | BC107009 |
|  | BC015613 |
|  | M68891 |
|  | AF169253 |
|  | NM\_008090 |
|  | AK004675 |
|  | NM\_032638 |
|  | AY032734 |
|  | BC061745 |
|  | BC051272 |
|  | BC018988 |
|  | AK154981 |
|  | AB000096 |
|  | BC015577 |
|  | AK154792 |
|  | AK004551 |
|  | M77810 |
|  | BC051342 |


---

|  |  |
| --- | --- |
| Protein | Q924Y4 |
|  | BAE32831 |
|  | AAH02557 |
|  | AAP35317 |
|  | AAA35869 |
|  | AAI07010 |
|  | AAH51272 |
|  | BAA19053 |
|  | O09100 |
|  | BAB23365 |
|  | P23769 |
|  | AAA35868 |
|  | AAH51342 |
|  | NP\_254277 |
|  | AAH15577 |
|  | AAK51128 |
|  | AAH61745 |
|  | AAH18988 |
|  | AAH15613 |
|  | AAL57180 |
|  | NP\_032116 |
|  | BAE32970 |
|  | BAB23463 |
|  | NP\_116027 |


---

|  |  |
| --- | --- |
| Organism | Mammal |


---

|  |  |
| --- | --- |
| Location | chromosome 6, 6 38.5 cM, 6 D1 (Mus musculus) |
|  | 6 38.5 cM (Mus musculus) |
|  | chromosome 3, 3q21.3 (Homo sapiens) |
|  | chromosome 4, 4q34-q41 (Rattus norvegicus) |


---

|  |  |
| --- | --- |
